# Electroacupuncture alleviates comorbid obesity and depression via the gut-brain axis: orchestrating SCFA-producing bacteria and hippocampal synaptic plasticity

**DOI:** 10.3389/fmicb.2026.1772788

**Published:** 2026-03-04

**Authors:** Yaxin Zhang, Yuxin Pang, Haoyuan Tan, Ronghui Xian, Junquan Liang, Qianyi Wen, Zhongxian Li, Luda Yan, Zeping Xie, Jingjing Li, Wenbin Fu, Peng Zhou

**Affiliations:** 1 Shenzhen Bao'an Traditional Chinese Medicine Hospital, The Seventh Clinical Medical School of Guangzhou University of Chinese Medicine, Guangzhou University of Chinese Medicine, Shenzhen, Guangdong, China; 2 Shenzhen Institutes of Advanced Technology, Chinese Academy of Sciences, Shenzhen, Guangdong, China; 3 Guangdong Provincial Hospital of Traditional Chinese Medicine, The Second Affiliated Hospital of Guangzhou University of Traditional Chinese Medicine, Guangzhou University of Chinese Medicine, Guangzhou, Guangdong, China

**Keywords:** depression, electroacupuncture, gut-brain axis, obesity, synaptic plasticity

## Abstract

**Introduction:**

Comorbid obesity and depression (COMBD) represents a complex metabolic-neuropsychiatric challenge with limited therapeutic options. While Electroacupuncture (EA) is effective for both metabolic and mood disorders, the systemic mechanisms—particularly the interplay between the gut microbiome and hippocampal plasticity—remain elusive.

**Methods:**

We established a COMBD rat model using a high-fat diet combined with chronic unpredictable mild stress (CUMS). An integrated multi-omics approach comprising 16S rDNA sequencing, LC-MS/MS serum metabolomics, and hippocampal transcriptomics was utilized to decipher the therapeutic mechanisms of EA.

**Results:**

EA treatment significantly attenuated body weight gain and reversed depressive-like behaviors. Crucially, EA restructured the dysbiotic gut microbiota, specifically increasing the abundance of short-chain fatty acid (SCFA)-producing bacteria. This microbial restoration was strongly correlated with a reprogrammed serum metabolic profile. In the hippocampus, transcriptomic analysis identified Cd74 as a pivotal upstream regulator modulated by EA. Furthermore, EA mitigated hippocampal oxidative stress and restored synaptic plasticity, evidenced by increased dendritic spine density and upregulated synaptic protein expression.

**Conclusion:**

Our findings suggest that EA ameliorates COMBD via a coordinated “Microbiota-Metabolism-Brain” axis. Specifically, EA creates a neuroprotective milieu by promoting beneficial SCFA-producing bacteria and regulating metabolic signals, which subsequently targets hippocampal Cd74 to restore synaptic plasticity. This study provides a novel mechanistic basis for the clinical application of EA in treating complex metabolic-mood comorbidities.

## Graphical abstract

**Figure:**
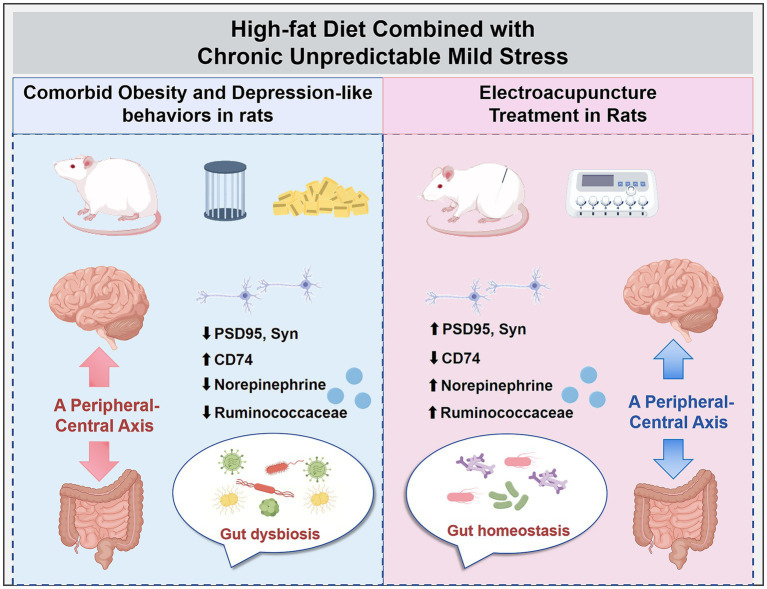

## 1 Introduction

Nowadays, obesity has lead to many complications, such as type 2 diabetes, hypertension, fatty liver and cardiovascular diseases (Koliaki et al., 2023). In particular, overweight/obesity and related complications are highly prevalent in the Chinese population, constituting a major public health challenge (Chen K. et al., 2023). Meanwhile, major depressive disorder (MDD) also has a significant impact on the global disease burden. Epidemiological evidence reveals a strong two-way relationship between these diseases. Even in the absence of any metabolic consequences, overweight can result in depression. There is a causal relationship between higher body mass index and lower glutamine levels. Both obesity and down regulation of glutamine have been causally linked to depression (Karageorgiou et al., 2023; He R. et al., 2023). This co incidence leads to a more serious disease trajectory, a worse responses to conventional treatment, and a significant increase in the risk of cardiovascular and metabolic diseases. The clinical management of comorbid obesity and depression (COMBD) is particularly challenging, as standard pharmacological treatments might yield unsatisfactory outcomes-some antidepressants can promote weight gain, while certain anti-obesity agents may adversely affect mood. The treatment of obesity can reduce the symptoms of comorbid depression, on the contrary, treatment of depression can improve the result of weight loss (Dębski et al., 2024). Therefore, developing safe and effective treatment strategies that can simultaneously solve metabolic and mental symptoms represents an urgent and unsatisfied medical need.

Electroacupuncture (EA) promotes weight loss, improves BMI, and regulates mood in both clinical and preclinical studies (Lam et al., 2024; Zhu et al., 2024; Yin et al., 2022). Its therapeutic effects are attributed to multi-target modulation of neuroendocrine circuits, inflammation, and energy metabolism (Wang M. N. et al., 2024). Despite empirical support, however, the mechanisms by which EA concurrently alleviates obesity and depression remain poorly understood. In particular, it is unclear how this peripheral intervention engages the central nervous system to correct the shared pathophysiology linking these two disorders.

The hippocampus is a brain region integral to emotional regulation, cognitive function, and energy homeostasis. It is vulnerable to chronic stress and metabolic insults, which are hallmarks of both obesity and depression. These factors converge to induce hippocampal damage, notably induce hippocampal damage by impairing synaptic plasticity (Tartt et al., 2022; Wang et al., 2021; Liśkiewicz et al., 2021). The origin of this central dysfunction may lie in the periphery. Clinical and animal studies have shown that disturbances in the gut microbiome can affect neural function and behavior through the microbiota-gut-brain axis, contributing to the pathogenesis of several brain diseases (Borsini et al., 2021). Recent evidence further suggests that peripheral metabolic disturbances, such as dyslipidemia and alterations in gut-derived metabolites like short-chain fatty acids and bile acids, can disrupt the central nervous system microenvironment. This leads to exacerbated oxidative stress and neuroinflammation, which in turn impair synaptic function (Huang et al., 2023). Therefore, a peripheral-central axis hypothesis is crucial for understanding COMBD. Yet, the specific metabolic factors involved, and how their modulation by EA influences hippocampal synaptic plasticity, are largely unknown. A comprehensive understanding requires an integrated approach to capture the complex interplay between systemic metabolism and central networks.

In this study, a multi-omics strategy to explore the therapeutic effects and potential mechanisms of EA in a rat model of COMBD was adopted. It hypothesized that EA would improve both metabolic phenotypes and depressive-like behaviors by restoring hippocampal synaptic plasticity. This study further proposed that this therapeutic effect is driven by a coordinated rewiring of the peripheral metabolome and the hippocampal transcriptome. Then this study aimed to characterize the gut microbial composition and the serum metabolic profile reshaped by EA using 16S rDNA sequencing and LC–MS/MS metabolomics, respectively. Additionally, the functional and structural improvements of hippocampal synapses and identify key upstream regulatory factors in the hippocampus through transcriptomics were evaluated. The research is designed to bridge the peripheral and central mechanisms of EA, providing a potential metabolic axis to explain its therapeutic effects on COMBD and identifying new potential targets for future therapies.

## 2 Methods and materials

### 2.1 Experimental design and animals

SPF male SD rats (weight 180–200 g) were purchased from Zhuhai baishitong biology science and technology co., ltd. All rats were housed under standard laboratory conditions (12-h light/dark cycle, 22 ± 2 °C, 50 ± 10% humidity) with free access to food and water. After 1 week of acclimatization, rats were randomly divided into three groups (*n* = 10): Control group (C): Fed a standard chow diet and handled normally without Chronic Unpredictable Mild Stress (CUMS) procedures. Model group (HC): Fed a High-Fat Diet (HFD) and subjected to CUMS to induce the COMBD phenotype. EA treatment group (HCE): Subjected to the same HFD + CUMS protocol as the HC group but received EA treatment during the intervention period.

### 2.2 Model establishment

HFD Model: Rats in the HC and HCE groups were fed a HFD (containing 60% kcal from fat, Guangdong Medical Experimental Animal Center) for 12 weeks to induce obesity and metabolic dysfunction. CUMS Model: Concurrently with the HFD feeding (from the 9th week onwards), rats in the HC and HCE groups were exposed to a CUMS protocol for 4 weeks. The CUMS procedures, administered randomly, included food/water deprivation, cage tilting, soiled cage, tail clipping, cold swimming, and reversed light/dark cycle, to induce depressive-like behaviors (Chen Y. et al., 2023). In the whole the experiment, the rats in HC group and HCE group continued for a high-fat diet.

### 2.3 EA treatment

EA treatment was performed over the final 4 weeks of the experimental period. Rats in the HCE group were gently immobilized in a specially designed holder and received acupuncture at bilateral Zusanli (ST36), Fenglong (ST40), Tianshu (ST25), and Zhongwan (CV12). Sterile stainless-steel needles were inserted to a depth of 5–7 mm and subsequently connected to an electroacupuncture apparatus. Electrical stimulation was delivered in disperse-dense wave patterns (2/15 Hz) at an intensity of 0.5 mA, which induced mild vibratory movements of the hind limbs without signs of distress. Each treatment session lasted for 30 min and was conducted once daily. Rats in the control and model groups were subjected to the same immobilization procedure but did not undergo needle insertion or electrical stimulation.

### 2.4 Behavioral tests

All behavioral tests were conducted during the final week of the experiment, in a dedicated quiet room, and video-recorded for blinded analysis. OFT: Each rat was placed in the center of a square open-field arena and allowed to explore freely for 5 min. The total distance traveled and average speed were analyzed using supermaze software. SPT: Rats were first trained to consume 1% sucrose solution. After 24 h of water and food deprivation, they were presented with two pre-weighed bottles, one containing 1% sucrose solution and the other tap water, for 24 h. Sucrose Preference (%) = Sucrose intake/(Sucrose intake + Water intake) × 100%. TST: Rats were suspended by their tails from a bracket using adhesive tape. The total 5-min test was recorded, and the cumulative immobility time was scored. An animal was considered immobile when it hung passively and motionless.

### 2.5 Sample collection and biochemical assay

After the behavioral tests, rats were fasted overnight and anesthetized. Blood samples were collected from the abdominal aorta. Serum was separated by centrifugation and stored at −80 °C for subsequent biochemical and metabolomic analyses. Fresh fecal samples were collected from the colon, immediately snap-frozen in liquid nitrogen, and stored at −80 °C for subsequent 16S rDNA sequencing. The liver, visceral fat (VAT), subcutaneous fat (SAT), and brown adipose tissue (BAT) were rapidly dissected, weighed, and either fixed for histology or snap-frozen. The brains were rapidly removed, and the part of hippocampus were dissected on ice for molecular biology, while the others were fixed for histology. Serum levels of triglycerides (TG) and total cholesterol (TC) were measured using Triglyceride assay kit and Total cholesterol assay kit (Nanjing Jiancheng Bioengineering Institute) following the instructions.

### 2.6 Histological staining

H&E Staining: Fixed liver and adipose tissues were embedded in paraffin, sectioned at 5 μm thickness, and stained with Hematoxylin and Eosin (H&E). Histopathological changes were observed under a tissue slice digital scanner, and adipocyte area was quantified using ImageJ software. Nissl Staining: Fixed hippocampal tissues were sectioned and stained with Nissl stain solution (Wuhan servicebio technology CO., LTD.). Golgi Staining: Fresh brain tissues were impregnated using the Servicebio GolgiStain Kit. Well-impregnated neurons in the hippocampal CA1 region were selected, and dendritic spine density was analyzed.

### 2.7 Immunofluorescence (IF)

For PSD95/Syn and CD74/NeuN double staining, paraffin sections were deparaffinized, antigens retrieved, blocked and incubated with primary antibodies: PSD95 (servicebio, 1:200), Synaptophysin (servicebio, 1:500), CD74 (servicebio, 1:800), NeuN (servicebio, 1:500) overnight at 4 °C, followed by incubation with secondary antibodies and appropriate TSA dyestuff. Nuclei were counterstained with DAPI. Images were acquired using a fluorescence microscope.

### 2.8 16S rDNA sequencing and analysis

Gut microbial DNA was extracted from the rats’ fecal samples. The V3-V4 region of the 16S rDNA gene was amplified and sequenced on an Illumina platform by Shanghai Biotree Biomedical Technology Co., Ltd. The resulting data were processed using QIIME2 to generate Amplicon Sequence Variants (ASVs). Alpha and beta diversity analyses were performed, and LEfSe was used to identify differentially abundant taxa.

### 2.9 Serum metabolomics analysis

Serum metabolites were analyzed using an LC–MS/MS system. Chromatographic separation was conducted on an Agilent 1,290 UPLC system with an ACQUITY UPLC BEH C18 column (2.1 × 150 mm, 1.7 μm). The mobile phase consisted of (A) water with 0.1% formic acid and (B) methanol/water (95:5). Mass spectrometry was performed on a SCIEX 6500 QTRAP+ mass spectrometer with an ESI source operating in MRM mode. The ion source parameters were as follows: curtain gas, 35 psi; ion spray voltage, ±4,500 V; temperature, 450 °C; ion source gas 1 and 2, 50 psi. Data were acquired and processed using SCIEX Analyst (v1.7.32) and BIOTREE Bio Bud (v2.0.3) software. The raw data were processed for peak alignment, retention time correction, and peak extraction. Multivariate statistical analyses, including OPLS-DA, were performed. Metabolites with VIP > 1.0 and ANOVA *P*-VALUE < 0.05 were considered differential metabolites. Pathway enrichment analysis was performed based on the GO and KEGG database.

### 2.10 Hippocampus transcriptomics detection

The total RNA was extracted from hippocampal tissues using TRIzol reagent. RNA integrity was checked, and sequencing libraries were constructed according to the ABclonal mRNA-seq Lib Prep Kit and sequenced on an Illumina/BGI platform. After quality control, clean reads were mapped to the reference genome using HISAT2 platform. Differential expression analysis was performed using the DESeq2 R package. Genes with |log2(FoldChange)| > 1 and adjusted *p*-value< 0.05 were considered DEGs. GO and KEGG pathway enrichment analyses of DEGs were performed, and a PPI network was constructed using the STRING database.

### 2.11 RT-qPCR

Total RNA from the hippocampus was reverse-transcribed into cDNA. RT-qPCR was performed using SYBR® Green Pro Taq HS qPCR Master Mix (Accurate Biology) on a Roche LightCycler® 480 real-time fluorescence quantitative PCR system. The mRNA expression levels of target genes (Nrf2, PSD95, Syn, Cd74) were normalized to *β*-actin and calculated using the 2^(−ΔΔCt) method.

### 2.12 Western blotting (WB)

Hippocampal tissues were lysed in RIPA buffer to extract total protein. Proteins were separated by SDS-PAGE and transferred to PVDF membranes. After blocking for 2 h, the membranes were incubated overnight at 4 °C with primary antibodies against PSD95(1:1500), Synaptophysin(1:1500), Hif1α(1:1000), Nrf2(1:1000), and *β*-actin(1:5000). After incubation with HRP-conjugated secondary antibodies, protein bands were visualized using an ECL detection system and quantified using ImageJ software.

### 2.13 Statistical analysis

All data are presented as the mean ± standard deviation (SD). Statistical analyses were performed using GraphPad Prism software. Differences between two groups were analyzed by two-tailed Student’s t-test. For comparisons among multiple groups, one-way analysis of variance (ANOVA) followed by Tukey’s *post hoc* test was applied. For body weight data measured repeatedly over time, two-way repeated measures ANOVA (factors: time, group, and their interaction) was used. A *p*-value of less than 0.05 was considered statistically significant.

## 3 Results

### 3.1 Electroacupuncture ameliorates metabolic phenotypes and depressive-like behaviors in COMBD rats

**Figure 1:**
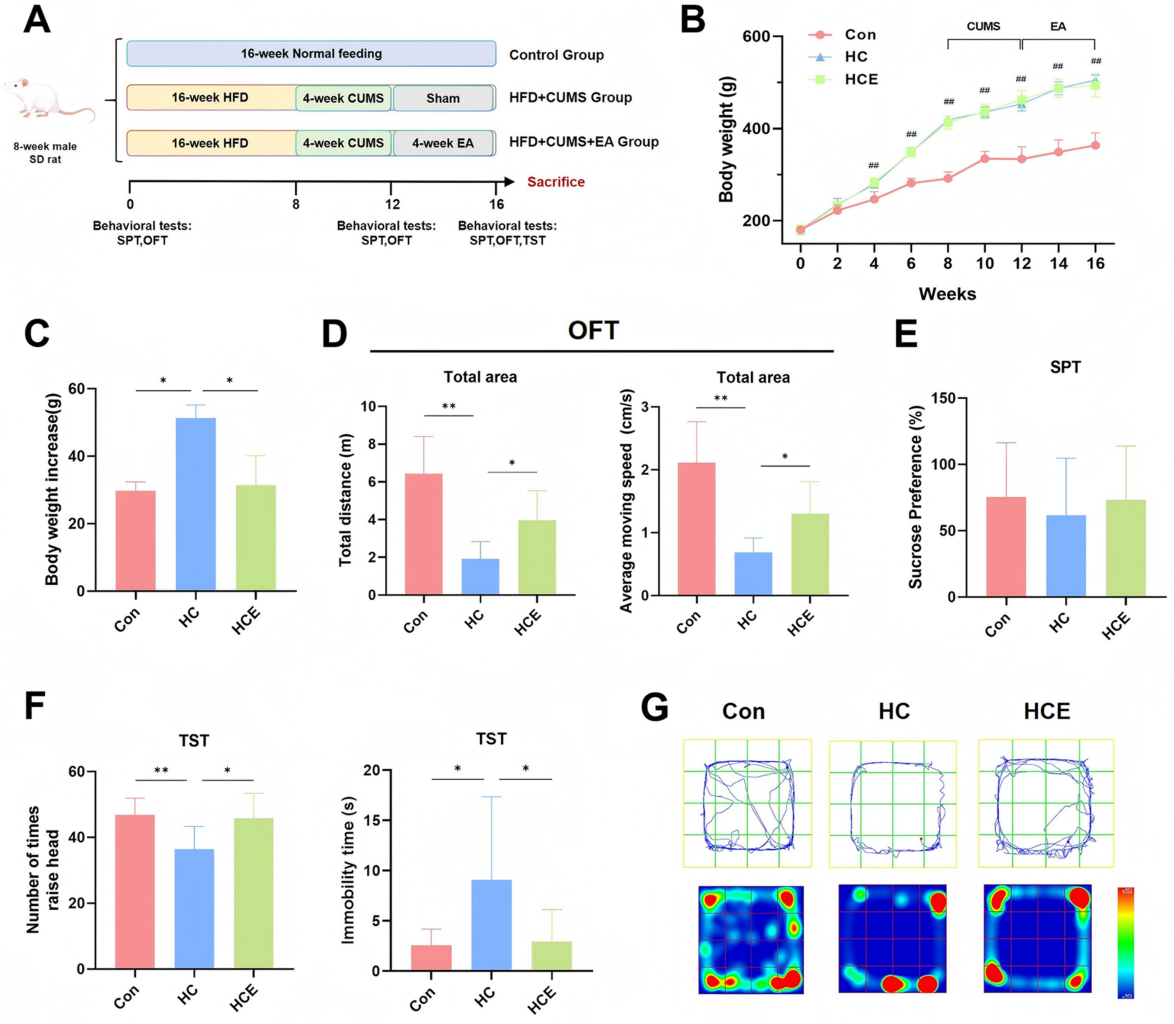
EA ameliorates metabolic phenotypes and depressive-like behaviors in COMBD rats. **(A)** Schematic diagram of the experimental timeline. **(B)** Body weight change curves of rats during the experiment. **(C)** Body weight gain of rats in each group during the 4-week EA intervention period. **(D)** Total distance and average speed in the Open Field Test in the 16th week. **(E)** Sucrose preference percentage in the Sucrose Preference Test. **(F)** Number of head lifts and immobility in the Tail Suspension Test. **(G)** Representative movement tracks and heatmaps from the Open Field Test. Data are expressed as mean ± standard deviation. One-way ANOVA was used to analyze statistical differences. Compared with model group, ^*^p < 0.05, ^**^p < 0.01. Compared with control group, ^#^*p* < 0.05, ^##^*p* < 0.01.

To investigate the therapeutic effect of EA on COMBD, we established a rat model and implemented an EA intervention regimen (Figure 1A). Compared to the control group, the high-fat diet/CUMS (HC) group exhibited a significant and sustained increase in body weight throughout the experimental period (Figure 1B). Notably, the body weight gain during the EA intervention period was significantly lower in the EA-treated (HCE) group compared to the HC group (Figure 1C). In behavioral assessments, COMBD rats showed markedly reduced locomotor activity in the OFT, as indicated by decreased total distance traveled and average speed (Figure 1D). While no significant difference in sucrose preference was observed in the SPT (Figure 1E), the HC group exhibited pronounced behavioral despair in the TST, characterized by a decreased number of head lifts and increased immobility time (Figure 1F). The movement tracks and heatmaps from the OFT visually confirmed the reduced exploration and central aversion in HC rats (Figure 1G). Critically, EA intervention significantly counteracted these behavioral deficits, restoring locomotor activity and active coping behavior. These results collectively demonstrate that EA effectively alleviates both metabolic and behavioral abnormalities in COMBD rats.

### 3.2 Electroacupuncture improves adipose tissue histopathology and lipid accumulation

**Figure 2:**
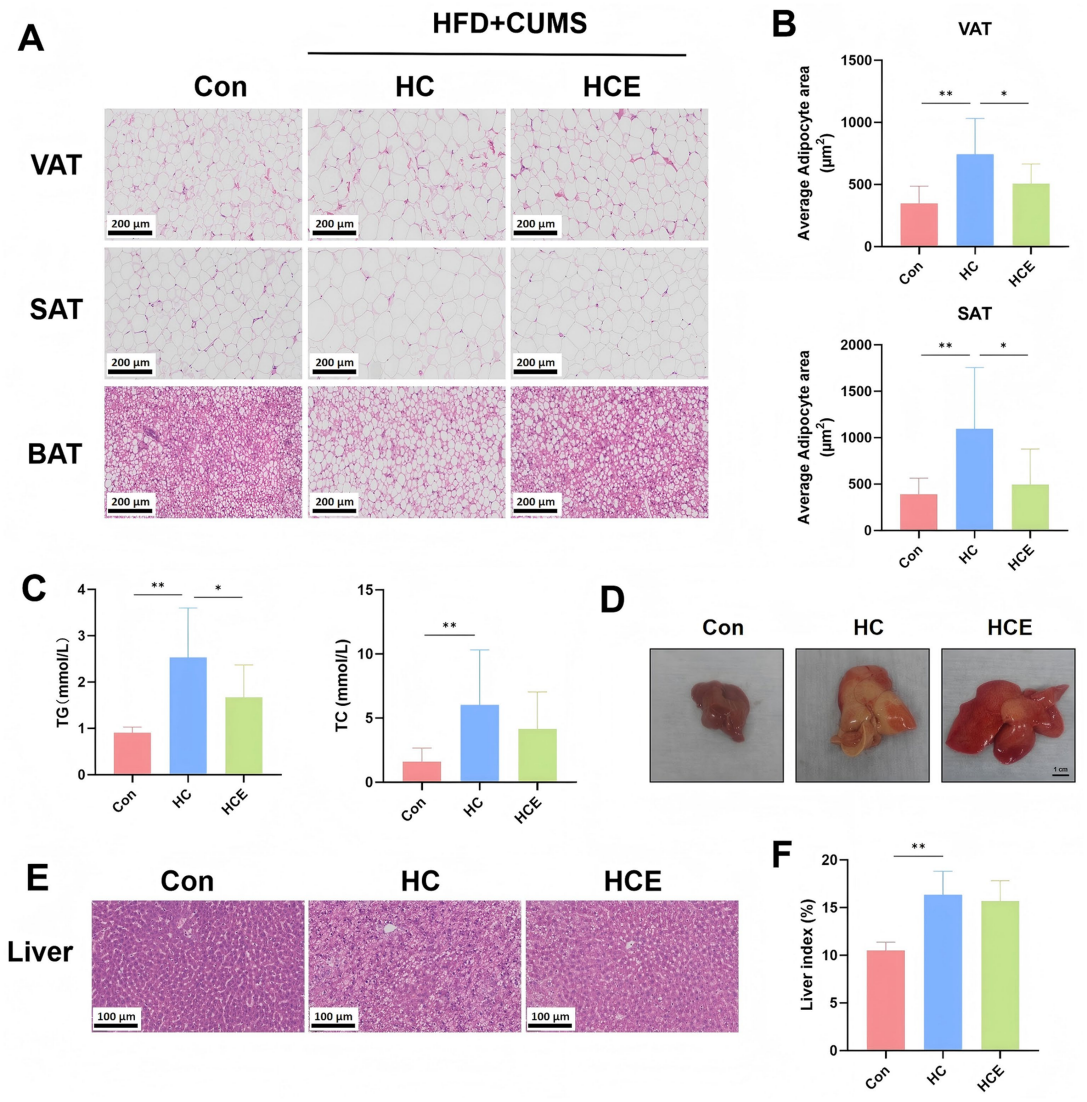
EA improves adipose tissue histopathology and lipid accumulation in COMBD rats. **(A)** Representative H&E-stained images of visceral adipose tissue (VAT), subcutaneous adipose tissue (SAT), and brown adipose tissue (BAT). **(B)** Quantitative analysis of adipocyte area in VAT and SAT. **(C)** Serum levels of triglycerides (TG) and total cholesterol (TC). **(D)** Representative macroscopic photographs of livers. **(E)** Representative H&E-stained images of liver tissue. **(F)** Liver index. Data are expressed as mean ± standard deviation. One-way ANOVA was used to analyze statistical differences. Compared with model group, ^*^p < 0.05, ^**^p < 0.01.

We next examined the impact of EA on peripheral metabolic organs and systemic lipid metabolism. Histological analysis of adipose tissue revealed that HC rats developed severe adipocyte hypertrophy in both visceral (VAT) and subcutaneous (SAT) fat depots, while EA treatment significantly reduced adipocyte size (Figures 2A,B). The morphology of brown adipose tissue (BAT) was also improved by EA (Figure 2A). Consistent with the histological improvements, EA significantly lowered elevated serum levels of TG (*p* < 0.05), although the reduction in TC did not reach statistical significance (Figure 2C). Macroscopic and microscopic examination of the liver showed that HC rats developed pronounced hepatic steatosis and an increased liver index. EA intervention significantly mitigated hepatic steatosis and exhibited a trend toward reduction in the liver index, though this reduction did not reach statistical significance (Figures 2D–F). These findings indicate that EA confers comprehensive benefits against obesity-related tissue pathology and systemic metabolic dysregulation.

### 3.3 Electroacupuncture alters gut microbiota composition in comorbid rats

**Figure 3:**
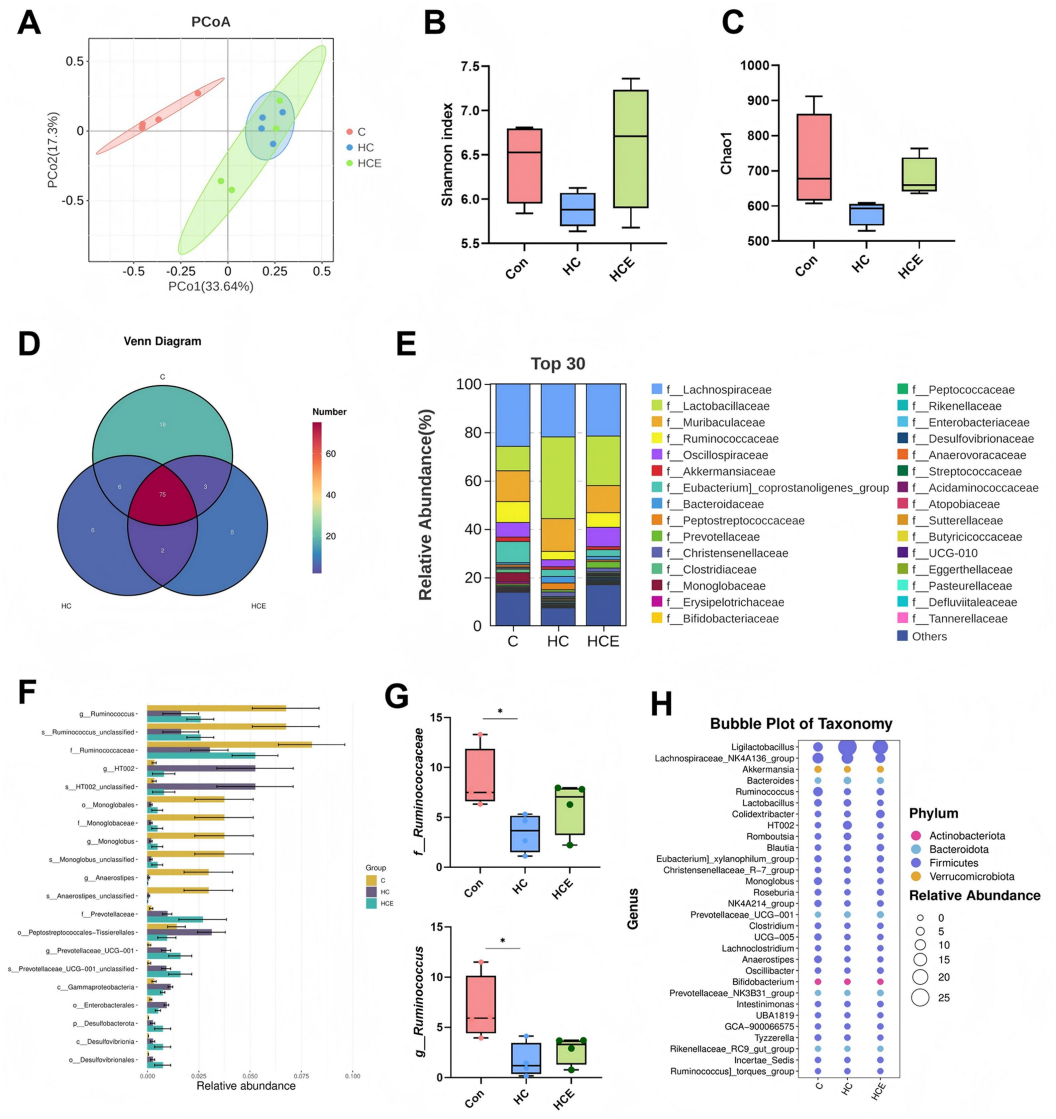
EA alleviates HFD/CUMS-induced gut dysbiosis. **(A)** PCoA plot based on Bray-Curtis distance shows the overall structural separation of the gut microbiota among groups. **(B,C)** *α*-diversity analysis of the gut microbiota, assessed by the Simpson index **(B)** and Chao1 index **(C)**. **(D)** Venn Diagram of the gut microbial composition at the family level. **(E)** Relative abundance of the gut microbial composition at the family level. **(F)** Cladogram generated from LEfSe analysis. **(G)** Relative abundance of the SCFA-producing family f__Ruminococcaceae and g_Ruminococcus. **(H)** Bubble plot visualizing the genus-level composition. The color of the bubbles represents the corresponding phylum (predominantly Firmicutes), and the size represents the mean relative abundance. *n* = 4.

The involvement of the gut microbiota in obesity and depression via the peripheral-central axis is increasingly recognized. To examine whether EA modulates the gut-brain axis, we conducted 16S rDNA sequencing on fecal samples. Principal coordinates analysis (PCoA) revealed a clear separation between the CON and HC groups, suggesting that gut dysbiosis was induced by HFD feeding and CUMS (Figure 3A). Both the Simpson and Chao1 indices were significantly decreased in HC rats, and these reductions in *α*-diversity were reversed after EA treatment (Figures 3B,C). Taxonomic analysis further illustrated compositional differences among the groups (Figures 3D,E). LEfSe analysis identified specific microbial biomarkers that distinguished each group (Figure 3F) and revealed EA-mediated remodeling of key bacterial genera, including the restoration of beneficial short-chain fatty acid (SCFA)-producing taxa such as those belonging to f__Ruminococcaceae (Figure 3G). The bubble plot indicated that several key differential genera mainly belonged to the phylum Firmicutes (Figure 3H). Collectively, these results demonstrate that EA effectively alleviates HFD and CUMS-induced gut dysbiosis.

### 3.4 Electroacupuncture significantly reshapes the serum metabolites

**Figure 4:**
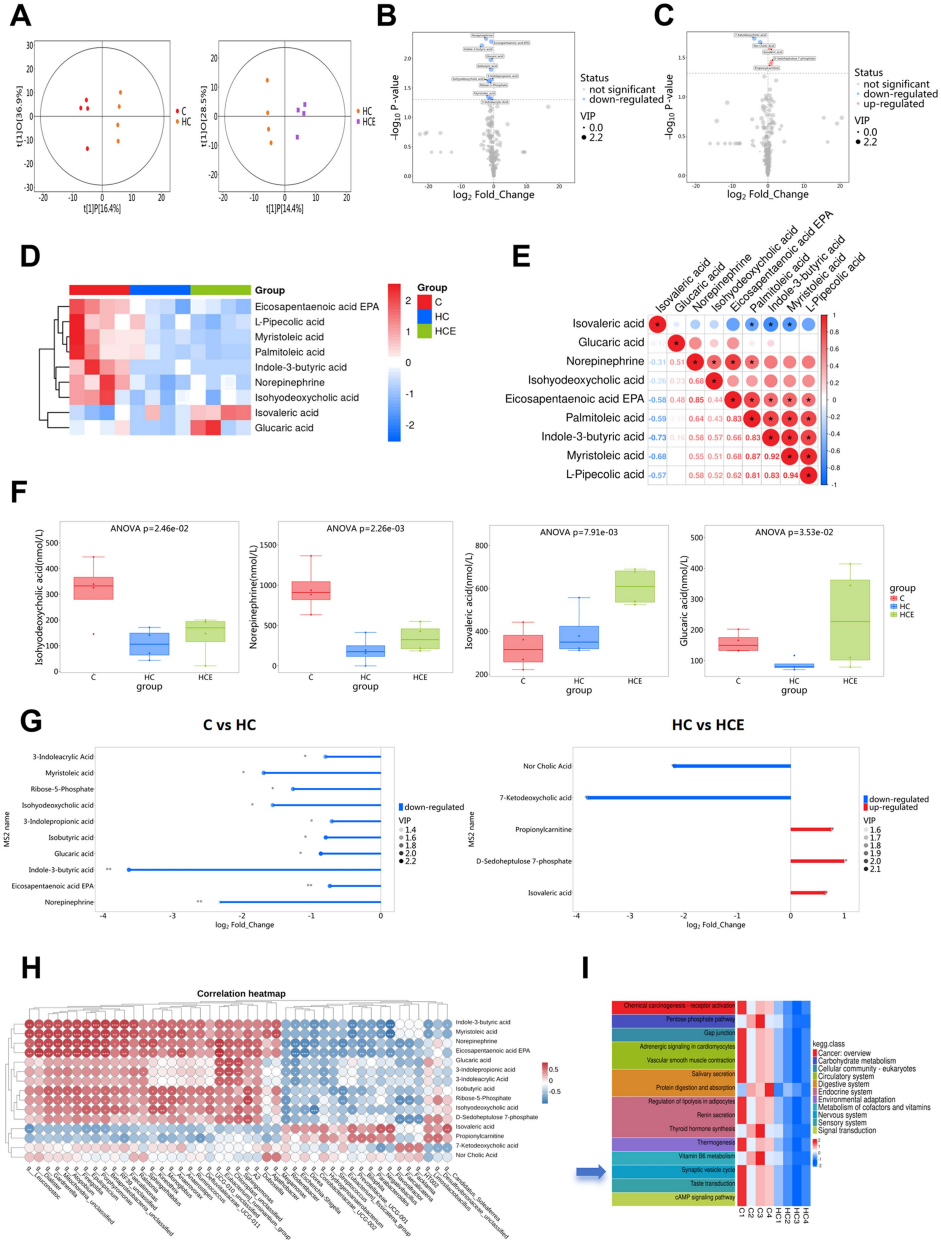
EA reshapes the serum metabolic profile based on metabolomics analysis. **(A)** OPLS-DA score plot showing separation of metabolic profiles among groups. **(B)** Volcano plot of differential metabolites between Group C and Group HC. **(C)** Volcano plot of differential metabolites between Group HC and Group HCE. **(D)** Hierarchical clustering heatmap of differential metabolites between Group HC and Group HCE. **(E)** Heatmap of correlation analysis for differential metabolites (HCE vs. HC). **(F)** Relative levels of key differential metabolites. **(G)** Matchstick plots for differential metabolites (C vs. HC and HC vs. HCE). **(H)** Heatmap of Pearson correlation analysis between differential gut microbial genera and differential serum metabolites. **(I)** KEGG pathway enrichment analysis of differential metabolites, suggesting regulation of pathways such as synaptic vesicle cycle. *n* = 4.

To investigate the microbiota-related metabolic alterations underlying the effects of EA, we performed serum metabolomic profiling. OPLS-DA revealed clear separations among the C, HC, and HCE groups, indicating distinct serum metabolic profiles (Figure 4A). Volcano plots identified differentially abundant metabolites in the C vs. HC and HC vs. HCE comparisons (Figures 4B,C). Unsupervised hierarchical clustering of these differential metabolites effectively distinguished the three groups, underscoring the substantial impact of EA on the serum metabolome (Figure 4D). Correlation and matchstick analyses further delineated specific metabolic shifts following EA treatment (Figures 4E,F). Among the most significantly altered metabolites, Isovaleric acid, D-Sedoheptulose 7-phosphate, and Propionylcarnitine were upregulated, whereas Norcholic Acid and 7-Ketodeoxycholic acid were downregulated in response to EA (Figure 4G). Subsequently, Pearson correlation analysis was conducted to explore relationships between differential gut microbes and metabolites. Indole-3-butyric acid, myristoleic acid, norepinephrine, and eicosapentaenoic acid (EPA) exhibited similar correlation patterns with differential bacterial taxa, while glucaric acid, 3-indolepropionic acid, and 3-indoleacrylic acid displayed another set of coordinated relationships (Figure 4H). KEGG enrichment analysis of the differential metabolites revealed a significant association with the ‘Synaptic vesicle cycle’ pathway, suggesting a potential link between peripheral metabolic changes and central synaptic function (Figure 4I). Together, these results suggest that EA induces a beneficial reprogramming of host metabolism, potentially initiating a gut microbiota-metabolite-brain signaling cascade.

### 3.5 Electroacupuncture fosters a pro-plasticity synaptic environment

**Figure 5:**
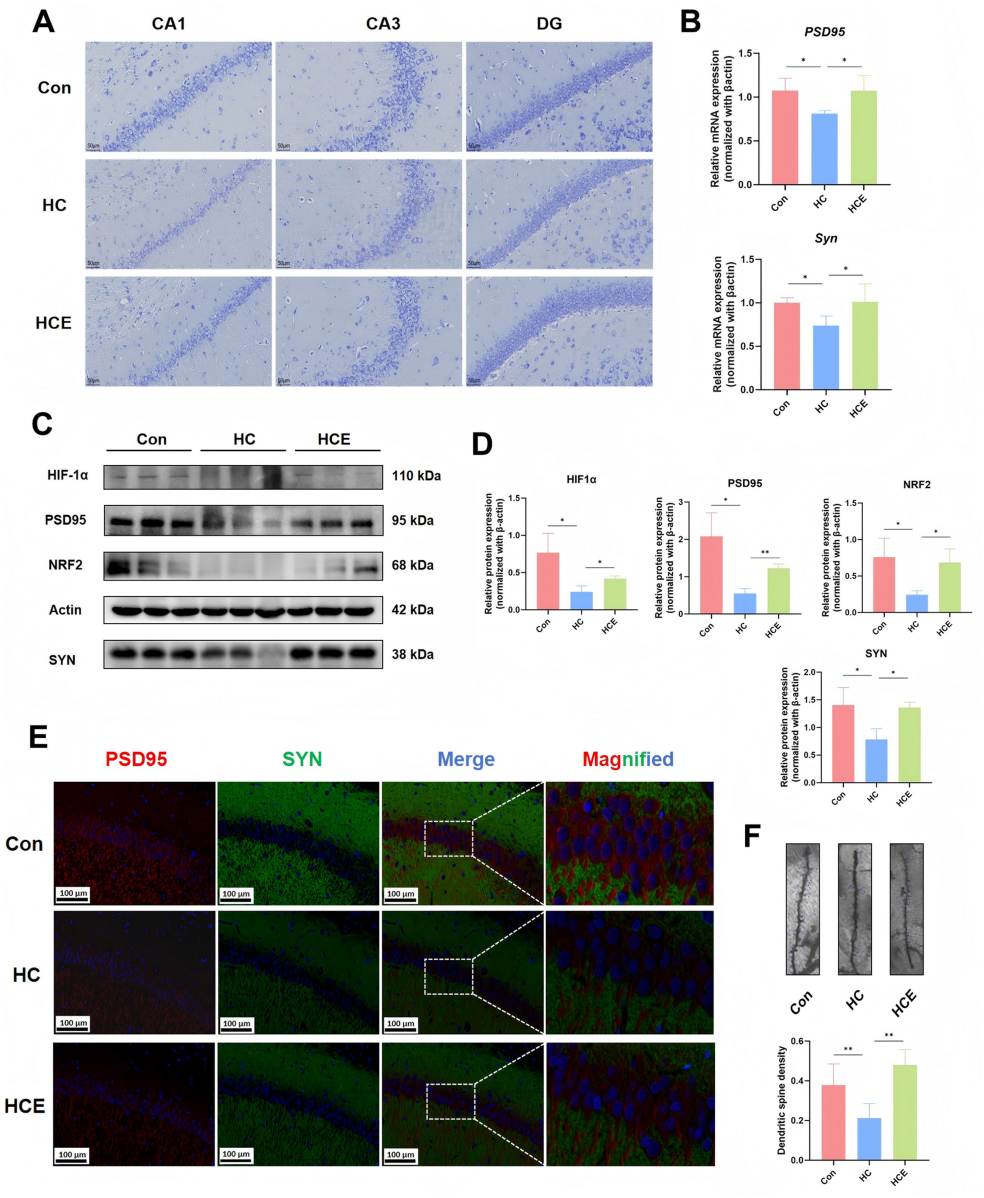
EA improves hippocampal synaptic plasticity by mitigates synaptic dysfunction and structure in COMBD rats. **(A)** Nissl staining of the hippocampal CA1, CA3, and DG regions. **(B)** mRNA expression levels of PSD95, and Syn in hippocampus. **(C)** Representative Western blot bands of oxidative stress-related proteins (HIF1α, NRF2) and synaptic plasticity proteins (PSD95, SYN) in the hippocampus. *n* = 3. **(D)** Quantitative analysis of Western blot. **(E)** Representative immunofluorescence images of double staining for PSD95 (red) and SYN (green) in the hippocampal region. **(F)** Golgi-stained hippocampal neurons, displaying dendritic spine morphology. *n* = 3. Data are expressed as mean ± standard deviation. One-way ANOVA was used to analyze statistical differences. Compared with model group, ^*^p < 0.05, ^**^p < 0.01.

Recent evidence indicates that gut proinflammatory bacteria are associated with abnormal functional connectivity of the hippocampus in unmedicated patients with major depressive disorder (Xiao et al., 2024). Based on this, we sought to investigate whether EA influences hippocampal synaptic plasticity. Nissl staining revealed that EA treatment attenuated neuronal damage in the hippocampal CA1, CA3, and DG regions of COMBD rats (Figure 5A). At the transcriptional level, EA up-regulated the expression of synaptic proteins PSD95 and Syn (Figure 5B). Western blot analysis further showed that EA elevated protein levels of the antioxidative stress markers Hif1α and Nrf2, while also robustly increasing the expression of PSD95 and Syn (Figures 5C,D). Immunofluorescence double staining confirmed enhanced expression and co-localization of PSD95 and Syn in the hippocampus following EA administration (Figure 5E). Moreover, Golgi staining indicated that EA significantly increased the density of dendritic spines, the primary sites of excitatory synapses, compared to the HC group (Figure 5F). It suggests that EA promotes a synaptic environment conducive to plasticity by mitigating oxidative stress and enhancing the expression of key synaptic proteins. Overall, our results demonstrate that EA not only preserves neuronal integrity in the hippocampus but also drives structural remodeling that supports improved synaptic plasticity.

### 3.6 Hippocampal transcriptomics identifies CD74 as a key mediator of EA-induced synaptic improvement

**Figure 6:**
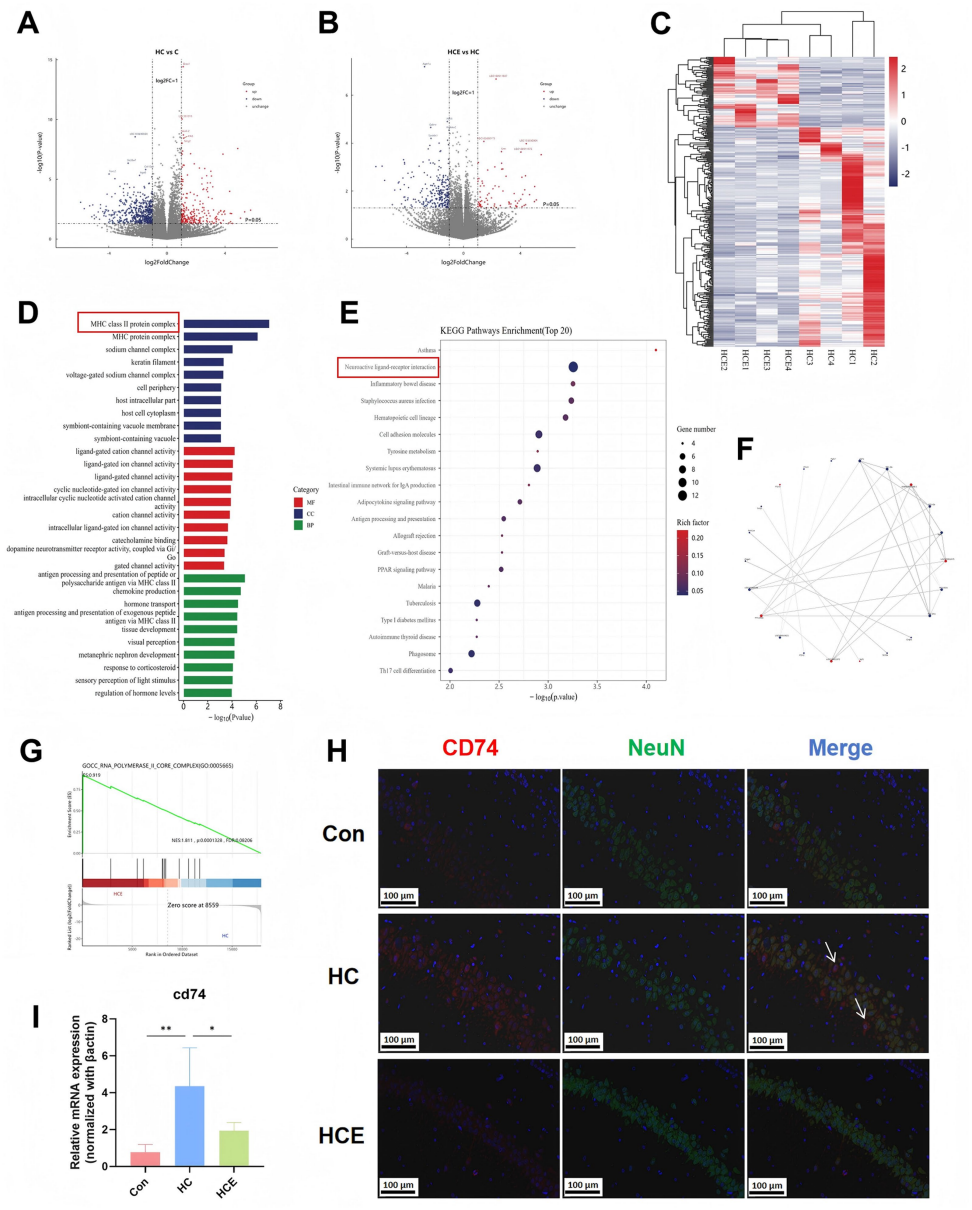
Hippocampal transcriptomics identified CD74 as a key molecule in EA-mediated synaptic plasticity improvement. **(A)** Volcano plot of differentially expressed genes (DEGs) between Group HC and Group C. **(B)** Volcano plot of DEGs between Group HCE and Group HC. **(C)** Clustering heatmap of DEGs between Group HCE and Group HC. **(D)** GO pathway enrichment analysis of DEGs, indicating significant enrichment in the MHC class II protein complex pathway. **(E)** Bubble plot of KEGG pathway enrichment analysis for DEGs. **(F)** PPI network circle of the top 30 confidence-scored DEGs. **(G)** GSEA enrichment for the MHC class II pathway. **(H)** Representative immunofluorescence images showing localization of CD74 (red) with the neuronal marker NeuN (green) in the hippocampus. **(I)** Cd74 mRNA expression level in hippocampal tissue. *n* = 4. Data are expressed as mean ± standard deviation. One-way ANOVA was used to analyze statistical differences. Compared with model group, ^*^p < 0.05, ^**^p < 0.01.

To elucidate the upstream molecular regulators, we conducted hippocampal transcriptomic analysis. Volcano plots and a clustering heatmap displayed differentially expressed genes (DEGs) between the comparison groups (Figures 6A–C). GO and KEGG enrichment analyses of these DEGs pointed to a highly significant involvement of the ‘MHC class II protein complex’ pathway and the ‘Neuroactive ligand-receptor interaction’ pathway (Figures 6D,E). Protein–protein interaction (PPI) network analysis identified key hub genes (Figure 6F), and Gene Set Enrichment Analysis (GSEA) confirmed the significant enrichment of the MHC class II pathway in the HCE group compared to HC (Figure 6G). Within this pathway, *Cd74* emerged as a top candidate. IF staining for CD 74 and NeuN was used to observe the expression of CD74 in hippocampal neurons (Figure 6H). RT-qPCR confirmed that EA also significantly down-regulated the mRNA expression of *Cd74* in the hippocampus (Figure 6I). These results suggest that CD74, a key molecule in the MHC class II pathway, serves as a potentially critical target through which EA modulates hippocampal synaptic plasticity in the COMBD state.

## 4 Discussion

A complex interplay exists between depression and obesity, where the two conditions can influence each other bidirectionally (Dębski et al., 2024). Consistent with EA’s multi-target effects, our study confirms its dual efficacy in a COMBD rat model. The acupoint prescription-bilateral Zusanli (ST36), Fenglong (ST40), Tianshu (ST25), and Zhongwan (CV12)-was selected based on traditional Chinese medicine theory and modern evidence. ST36 regulates energy metabolism and inflammation (Chen et al., 2025; Liu et al., 2021); ST40 resolves “phlegm-dampness” and influences lipid metabolism (Jin et al., 2023; Xue et al., 2021); ST25 modulates gut motility and visceral fat; and CV12 harmonizes gastrointestinal function to facilitate gut-brain communication (Tang et al., 2020; He Y. et al., 2023). The antidepressant effects of ST36 and ST25 are linked to regulating gut microbiota and neurotransmitter systems (Wang J. et al., 2024). Together, these acupoints form a synergistic network that addresses both metabolic and emotional dysregulation, providing a holistic therapeutic approach ideally suited for COMBD.

One of the key finding is EA-induced systemic metabolic reprogramming. Serum metabolomics revealed modulation of gut microbiota-derived metabolites. Specifically, EA restored SCFA-producing Ruminococcaceae, which is often depleted in obesity and depression (Radjabzadeh et al., 2022; Hui et al., 2022). Reduced Ruminococcaceae is linked to impaired SCFA production and systemic inflammation (Cao et al., 2025; Cornejo-Pareja et al., 2024). Pearson correlation analysis showed strong associations between EA-modulated gut microbiota and norepinephrine, suggesting gut microbiota may regulate peripheral norepinephrine levels, thereby influencing central functions related to motivation and stress (Kim et al., 2025). Isovaleric acid, which ameliorates chronic stress and inflammation by inhibiting NF-κB (Guo et al., 2025), correlates negatively with obesity markers (Ostrowska et al., 2024). EA also modulated bile acids like deoxycholate, which increase in metabolic liver disease (Smirnova et al., 2022). Pathway analysis linked these serum metabolic changes to synaptic plasticity-related pathways, indicating that EA creates a healthier peripheral metabolic environment that supports central recovery.

Guided by metabolic findings, we examined hippocampal changes. The KEGG enrichment analysis of the differential metabolites revealed a significant correlation with the synaptic vesicles cycle pathway. PSD95 and synaptophysin protein levels were used as indicators of synaptic plasticity in hippocampus (Shahar et al., 2024). The down-regulation of HC group and the up-regulation of HCE PSD95 and Syn expression indicate that the enhanced synapsis is relieved. The immunoco-localization of these synaptic proteins further supports enhanced synaptic connectivity (Silva et al., 2024). It also shows the regulation of Hif1α and Nrf2, which indicates that oxidative stress of HCE group is improved. Dynamic changes and functions of the HIF and NRF2 signal pathways play an important role under hypoxia and oxidative stress (Bae et al., 2024). Nrf2^−/−^ mice showed impaired FC in limbic system and the basal ganglia, especially in the hippocampus. Inhibition of iron accumulation effectively weakened CUMS-induced synaptic damage, which was mediated by down-regulating brain-derived neurotrophic factor. Activation of Nrf2 restored iron homeostasis, and reversed vulnerability to depression (Zeng et al., 2023). Therefore, we assume that the improvement in peripheral metabolism, especially the reduction of pro-oxidant metabolites, will help reduce the oxidative load of hippocampus, thus creating a licensed environment for synaptic plasticity.

Although body weight may act as a confounding factor, the pronounced reversal we observed at the molecular level, in particular the synaptic proteins and transcriptomic profiles, indicates that the recovery of neuroplasticity is not merely a by-product of weight loss. The direct morphological evidence further supports the functional improvements at the molecular level. Synaptic plasticity in the hippocampal region is known to be impaired in HFD-induced depression-like behavior (Wu et al., 2024). In this study, we assessed dendritic spine density in the hippocampus using Golgi staining. EA treatment markedly increased dendritic spine density, providing a structural basis for enhanced synaptic communication (Yin et al., 2023). Together, the stepwise changes observed, from metabolic and protein alterations to structural remodeling, suggest that EA-induced biochemical adaptations create a permissive environment for the reorganization of hippocampal neuronal circuits.

In order to identify upstream regulatory events, this study turned to hippocampus transcriptomics. The identification of CD74 as a key target is particularly intriguing. While traditionally recognized for its role in immune signaling as the receptor for MIF, its involvement in synaptic plasticity and neuroinflammation represents an emerging concept (Hong et al., 2024; Shishkina et al., 2023). Our demonstration of CD74 expression in the hippocampus, combined with pathway analysis implicating the MHC class II pathway, suggests EA may modulate a neuro-immune interface via CD74, subsequently influencing downstream pathways controlling oxidative stress response and synaptic protein synthesis. Thus, CD74 emerges as a crucial transcriptional node through which EA orchestrates its pleiotropic effects on hippocampal plasticity in the COMBD state.

Although we observed significant behavioral improvements, the present study mainly demonstrates associations rather than direct causality. Future studies utilizing more specific antagonists or genetic approaches will be required to further disentangle the respective contributions of weight loss and neuroplasticity to the behavioral outcomes. In addition, an important limitation is that the mechanisms by which peripheral metabolites influence hippocampal function remain incompletely understood, and the role of CD74 is supported only indirectly. Functional gain-of-function and loss-of-function studies targeting CD74 are needed to establish its causal involvement in linking peripheral metabolic changes to hippocampal plasticity. Future research should therefore focus on clarifying how specific metabolites engage the hippocampal CD74 pathway and mediating downstream neuroplastic changes.

## 5 Conclusion

In summary, our study elucidates that electroacupuncture exerts therapeutic effects against comorbid obesity and depression through a multi-tiered axis. EA initiates its action in the periphery by restoring gut microbial homeostasis, specifically enriching SCFA-producing taxa like Ruminococcaceae. This microbial remodeling is accompanied by a recalibration of the serum metabolome, characterized by elevated levels of beneficial metabolites such as isovaleric acid. These peripheral signals likely influence the hippocampus to mitigate oxidative stress, downregulate CD74, and enhance synaptic plasticity. Our findings provide a scientific basis for using EA to treat metabolic-psychiatric comorbidities and reveal novel microbial and metabolic targets for therapeutic development.

## Data Availability

The datasets presented in this study can be found in online repositories. The names of the repository/repositories and accession number(s) can be found at: NCBI BioProject PRJNA1423444 (https://www.ncbi.nlm.nih.gov/bioproject/PRJNA1423444).
